# Clinical Feature of Intrahepatic B-Lymphocytes in Chronic Hepatitis B

**DOI:** 10.1155/2014/896864

**Published:** 2014-01-21

**Authors:** Ashraf Mohamadkhani, Elnaz Naderi, Masoud Sotoudeh, Aezam Katoonizadeh, Ghodratollah Montazeri, Hossein Poustchi

**Affiliations:** Liver and Pancreatobiliary Diseases Research Center, Digestive Diseases Research Centre, Tehran University of Medical Sciences, Shariati Hospital, North Kargar Avenue, Tehran 14117 13135, Iran

## Abstract

Humoral immunity constitutes major defense mechanism against viral infections. However, the association of hepatic injury and B-cells population in chronic hepatitis B virus (HBV) carriers has not been studied well. In this study, fifty seven hepatitis B surface antigen (HBsAg) positive and HBeAg negative patients were studied to determine the expression of CD20, a cell surface marker expressed on B-cells, in liver biopsy sections using immunohistochemistry. The patients' clinical data at the time of liver biopsy were acquired from their medical records. There was a significant association between log HBV DNA and both ALT (*r* = 0.36, *P* = 0.006) and histologic activity index (HAI) total score (*r* = 0.3, *P* = 0.02), respectively. The CD20 was expressed in all 57 liver biopsy samples with a submembranous and membranous staining pattern and its expression was significantly associated with HAI total score (*r* = 0.32, *P* = 0.01) and stage of fibrosis (*r* = 0.31, *P* = 0.02). The susceptible B lymphocytes to hepatitis B virus might be implicated in the development of immune mediated inflammation of HBV-induced hepatic injury. The present data also support that the liver is potentially one of the secondary lymphoid organs.

## 1. Introduction 

Chronic hepatitis B (CHB) virus (HBV) infection is the principal cause of cirrhosis and hepatocellular carcinoma (HCC) [1]. The pathogenesis of HBV-related chronic liver disease is not well understood. However, it is clear that the immune mechanisms associated with the antiviral response are responsible for CHB outcome [2–4]. The existence of lymphocytes in the human liver is representing a pathological situation [5]. This concept stems from the observation that, in chronic hepatitis B, T-cells can potentially participate both in the immune clearance of HBV-infected cells and in the pathogenesis of hepatocellular injury [6]. Furthermore the numbers of B lymphocytes and plasma cells are significantly higher in patients with liver cirrhosis than of those with inactive chronic hepatitis [7, 8]. Enormous intrahepatic B-cells with massive production of IgM and IgG and infiltrating plasma cells into the hepatic lobules have also been shown in HBV-associated chronic active hepatitis [9]. B-cells contribute to immune responses through the secretion of effector cytokines and it has been suggested that naive and memory B-cell subsets preferentially produce different effector cytokines [10, 11].

Naïve B-cells undergo maturation by somatic hypermutation in immunoglobulin variable region of the B-cell receptor (BCR) genes following contact with a specific protein accessible on dendritic cells. Then the high affinity antigen receptors which normally consist of two isotypes membranes IgM and IgD continue to mature to either Ig-secreting plasma cells or memory B-cells [12]. In comparison to antigen primary response, immunological memory presents the capacity to increase a faster and more vigorous humoral response subsequent to antigen re-exposure [13]. Although antibody associated mechanisms targeting hepatitis B core antigen (HBcAg) was reported in earlier studies, few data exist on B lymphocytes population in the liver of patients with CHB. Cell markers are unique to identify and classify cell types. CD20 is a B-cell specific surface antigen that is expressed in all stages of B-cell development except on either early pro-B-cells or plasma cells and plays an important role in B-cell activation and proliferation [14].

To elucidate the role of intrahepatic B-cells in the pathogenesis of chronic hepatitis B, we investigated the expression of CD20 marker on B-cells in liver biopsy of these patients by immunohistochemistry.

## 2. Material and Methods

### 2.1. Patients

Liver biopsy specimens from 57 patients with HBV-associated chronic liver disease without liver neoplasm attending the Hepatitis Clinic of Shariati Hospital, Tehran University of Medical Sciences, during the years of 2008 to 2011 were studied. HBV infection was diagnosed by the positivity for hepatitis B surface antigen (HBsAg) in the patients' sera. All the patients were HBeAg negative and had a history of familial HBV infection, without coinfection with human immunedeficiency virus (HIV) or other hepatitis viruses. None of the patients had autoimmune hepatitis or other liver related diseases. The patients' clinical data at the time of liver biopsy were acquired from their medical records. No patients received anti-HBV therapy prior to liver biopsy. The protocol for this study was approved by the Ethics Committee of Shariati Hospital.

### 2.2. Histological Studies of Livers

The presence of CHB, stage of fibrosis, and histological activity were evaluated by modified histologic activity index (HAI) scoring system [15] on the liver sections stained with hematoxylin-eosin and Sirius red.

### 2.3. Immunohistochemistry and Analysis of Liver Biopsy Specimens

Commercially available primary monoclonal antibody against CD20 (clone UCHT1, Dako) was used to stain 3.0 *μ*m sections of liver biopsies after deparaffinization by routine microwave antigen retrieval and horseradish peroxidase (diaminobenzidine) kit (DAKO, Carpinteria, CA), following manufacturer's instructions. Immunohistochemical scoring ranged from 1 to 5 score (matched for 0–10%, 10–25%, 25–50%, 50–75%, and >75% CD20 positive cells) with higher scores indicating a greater proportion of positive cells (as determined from the positive control). For enumeration of CD20 positive B-cells, image acquisition was done with the +10, +20, and +40 objectives, but stained cells were counted in the entire field of +40 corresponding to a tissue area of 30 mm^2^. Positive staining for CD20 was done on the cytoplasmic side of the cell membrane. Positive stained cells appeared with no clear plasmacytoid morphology, but with granular cytoplasm and lobated nucleus of B-cells. A section from lymph node was used as a positive control for CD20 antigen.

### 2.4. Statistical Analysis

All the data were expressed as the mean ± standard deviation (SD). Correlations between variables were analyzed using the Pearson correlation coefficient (*r*) or Spearman rank correlation coefficient (*R*), as appropriate. A *P* value of <0.05 was deemed statistically significant.

## 3. Results 

### 3.1. Patients' Characteristics

Fifty-seven HBeAg negative patients were included in the present study. The baseline demographics are shown in Table 1. The mean ± SD age of patients was 33 ± 9 years and 40 (70%) were male. The mean ± SD of total HAI score for fibrosis and necroinflammation of patients is shown in Table 1. Nine patients (15.7%) had significant fibrosis more than or equal to 3.

### 3.2. Clinical Findings

To analyze the impact of B-cell population in CHB patients, the CD20 expression score, age, gender, log viral load, serum ALT, and HAI total score of necroinflammation and fibrosis were included in a multivariable-adjusted logistic regression model. There was a significant association between log HBV DNA with both ALT and HAI total score (*r* = 0.36, *P* = 0.006, and *r* = 0.3, *P* = 0.02), respectively. Serum ALT also showed positive correlation with total score of HAI though which was not significant (*r* = 0.25, *P* = 0.06). There were no lines of evidence of interactions and multicollinearity between variables age and sex with log HBV DNA and ALT. No significant correlation was observed between expression of CD20 B-cell marker expression with serum ALT and HBV DNA level.

### 3.3. Immunohistochemical Examination for Prevalence of B-Cells in Liver

**Figure 1:**
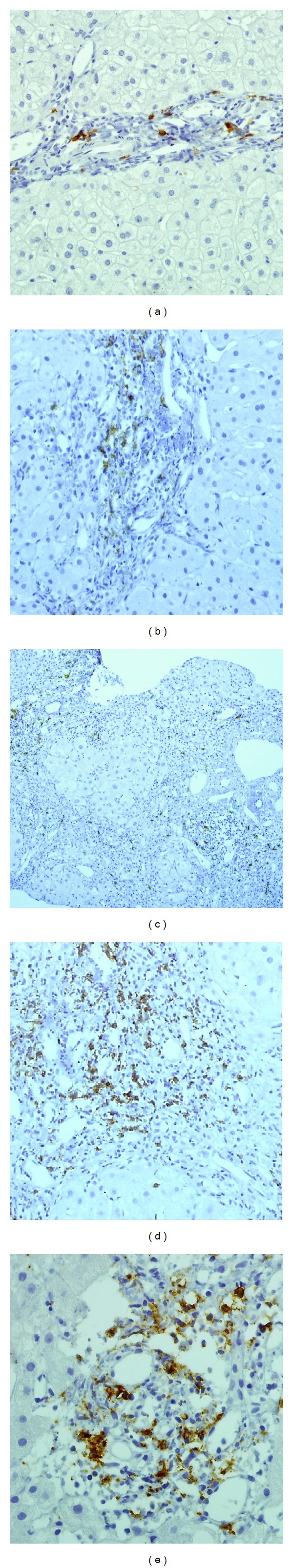
Representative microphotographs of CD20 marker by immunohistochemistry in hepatitis B virus-associated chronic liver diseases. ((a), (b)) Expression of CD20 in chronic hepatitis B patients without fibrosis and low fibrosis (original magnification, 200). ((c), (d), (e)) CD20 immunoreactivity was observed throughout the portal region of high fibrosis chronic hepatitis B patients (original magnification, 100, 200, and 400).

The liver was diffusely infiltrated by CD20 positive B-cells, distributed both as aggregates in the portal areas and as single cells within the lobules. The CD20 positive B-cells were expressed in all 57 liver biopsies with a submembranous and membranous staining pattern (Figure 1). The expression of CD20 was significantly associated with HAI total score (*r* = 0.32, *P* = 0.01) and stage of fibrosis (*r* = 0.31, *P* = 0.02). CD20 positive B-cells appeared as small clusters. Fifteen cases had score 1, thirty one had score 2, ten had score 3, and one had score 4 CD20 positive B-cells.

## 4. Discussion 

In this study, the role of intrahepatic B-cells in the pathogenesis of CHB infection has been investigated. The immunohistochemical findings of liver biopsies in this study indicate a major role for B-cells in the pathogenesis of CHB which is driven by increased CD20 positive B-cells population. Our evidence showed that CD20 positive B-cells clustered in the portal areas or as single cells within the lobule and correlated with necroinflammation and fibrosis which was consistent with previous studies [9]. Indeed our histological findings resembled the histological picture seen in patients with acute hepatitis B induced liver failure with a reflective B-cell response that could be responsible for huge expression of antibody to HBV core protein (HBcAb). However, few studies have reported this content. Our study indicates that B-cells are involved in inflammatory reactions for destruction of infected cells. Thus, they play an important role in the immune response of CHB infection.

Unlike HBsAg and HBeAg, the immunogenicity of HBcAg is able to directly activate B-cells to produce specific antibodies in an extralymphatic situation and in the absence of T-cells [9]. Moreover, it has been shown that the activation of B-cells, in a T-cell independent pathway, produced immunoglobulins and cytokines in the liver that changed fibrotic responses [11, 16]. Our recent study showed the association of IgD positive B-cells with the fibrosis stage in chronic hepatitis B [17]. The intrahepatic lymphocytes are recognized as long-lived recirculating B-cells and are similar to splenic B2 cells that produce cytokines IL-2, IL-4, TNF*α*, and IL-6 with proinflammatory function and contribute to disease pathogenesis in an antibody-independent fashion, perhaps by modulating T cell responses [16, 18]. Earlier studies revealed that B-cells evidently contribute to the development of liver fibrosis by the production of profibrotic cytokine IL-6 that induces the differentiation of hepatic stellate cells into myofibroblasts and raise collagen synthesis [16, 18, 19]. Both mature B lymphocytes in liver that belong to B2 subset [16] and resting B-cells typically have CD20 receptor [14]. Therefore, we could suggest CD20 marker, as a representative marker of both naive and preswitch memory B-cells.

Evaluation of intrahepatic B-cells population is important to determine how B-cells would respond to HBV infection. In this setting, evidence from the current study indicates that exposure to viral antigens can increase the proportion of B-cells in the liver to carry an exhausted phenotype and therefore to dysfunction. Our present data also support the property of liver as an immune system, which may potentially affect the pathogenesis of various liver diseases.

## Conflict of Interests

The authors declare that there is no conflict of interests regarding the publication of this paper.

## Figures and Tables

**Table 1:** Descriptive statistics of clinical and pathological variables in 57 chronic hepatitis B patients.

Variables	*N*	Minimum	Maximum	Mean	Std. deviation
Age (Years)	57	21	55	33	9
log HBV DNA (Copies/mL)	57	1.19	6.30	3.05	1.21
ALT (IU/L)	57	18	132	40	24
HAI score for total necroinflammation and fibrosis	57	1	9	3.65	1.54
Stage of fibrosis	57	0	5	1.61	1.031
Liver CD20 (score)	57	1	4	1.95	0.71
Liver IgD (score)	57	1	4	1.95	0.91
